# Decursinol Protects Against Lipopolysaccharide-Induced Placental Inflammation and Trophoblast Dysfunction via Mitochondrial Preservation and NLRP3 Inflammasome Inhibition

**DOI:** 10.3390/cells15141309

**Published:** 2026-07-22

**Authors:** Solji Lee, Hye-ji Lee, Jiha Shin, Sohee Lee, Jaeku Kang, Seok-Rae Park, Jong-Seok Kim, Jongdae Shin, Tae-Eun Jin, Nam-Kyung Lee, Ju-Young Park, Jeong Sig Kim, Nak Song Sung, Sung Ki Lee, Hwan-Woo Park

**Affiliations:** 1 Department of Cell Biology, Konyang University College of Medicine, Daejeon 35365, Republic of Korea; leesolji0116@hanmail.net (S.L.); amyamy7982@gmail.com (H.-j.L.); dixest0337@gmail.com (J.S.); soheelee1854@gmail.com (S.L.); jskim7488@konyang.ac.kr (J.-S.K.); shinjd@konyang.ac.kr (J.S.); 2 Department of Pharmacology, Konyang University College of Medicine, Daejeon 35365, Republic of Korea; jaeku@konyang.ac.kr; 3 Department of Microbiology, Konyang University College of Medicine, Daejeon 35365, Republic of Korea; srpark@konyang.ac.kr; 4 Myungok Medical Research Institute, Konyang University, Daejeon 35365, Republic of Korea; sklee0728@konyang.ac.kr; 5 Korea Bioinformation Center, Korea Research Institute of Bioscience and Biotechnology, Daejeon 34141, Republic of Korea; tejin@kribb.re.kr; 6 Biotherapeutics Translational Research Center, Korea Research Institute of Bioscience and Biotechnology, Daejeon 34141, Republic of Korea; nklee@kribb.re.kr; 7 MabTics Co., Ltd., Daejeon 34141, Republic of Korea; 8 CUREVEX Co., Ltd., Chungju 27478, Republic of Korea; pink1209juyoung@empas.com; 9 Department of Obstetrics and Gynecology, Soonchunhyang University Seoul Hospital, Seoul 04401, Republic of Korea; jskim@schmc.ac.kr; 10 Department of Surgery, Konyang University Hospital, Daejeon 35365, Republic of Korea; kysns@konyang.ac.kr; 11 Department of Obstetrics and Gynecology, Konyang University Hospital, Daejeon 35365, Republic of Korea

**Keywords:** decursinol, trophoblast, placenta, NLRP3 inflammasome, mitochondrial ROS, pregnancy complication

## Abstract

**Highlights:**

**What are the main findings?**

- Decursinol suppresses LPS-induced NF-κB/NLRP3 inflammasome activation and inflammatory cytokine expression in trophoblasts and placental tissues.
- Decursinol reduces mitochondrial oxidative stress and preserves mitochondrial homeostasis, trophoblast invasion, and fetal–placental outcomes in an LPS-induced pregnancy complication model.

**What is the implication of the main finding?**

- Decursinol targets multiple nodes of placental inflammatory signaling, including NF-κB priming, NLRP3 assembly, and mitochondrial stress, making it a mechanistically distinct candidate for inflammation-driven pregnancy complications.
- These findings provide a preclinical rationale for further investigation of decursinol as a therapeutic agent in pregnancy complications characterized by excessive placental inflammation and mitochondrial stress.

**Abstract:**

Inflammation-induced placental dysfunction is a major contributor to pregnancy complications. Activation of NF-κB and NLRP3 inflammasome pathways in the placenta is a key driver of this pathology. Decursinol, a natural coumarin derivative from Angelica gigas, possesses anti-inflammatory properties; however, its effect on placental inflammation remains unclear. Therefore, in this study, we investigated the protective effects of decursinol against lipopolysaccharide (LPS)-induced placental inflammation and trophoblast dysfunction and explored the underlying molecular mechanisms. Decursinol significantly inhibited LPS-induced NLRP3 inflammasome activation and NF-κB/p65 signaling in Sw.71 human trophoblast cells, reducing interleukin-1β secretion and pro-inflammatory gene expression. It restored the trophoblast invasive capacity and preserved mesenchymal marker expression suppressed by LPS. It also improved the fetal and placental weights, restored the placental architecture, and attenuated placental NLRP3 inflammasome activation and cytokine expression in vivo. Mechanistically, decursinol preserved the mitochondrial homeostasis, reduced mitochondrial reactive oxygen species levels, and upregulated antioxidants and mitochondrial biogenesis-related gene levels, exerting effects comparable to those of mitochondria-targeted antioxidant Mito-TEMPO. These findings suggest that decursinol protects against LPS-induced trophoblast dysfunction and adverse pregnancy outcomes by preserving mitochondrial functions and suppressing NLRP3/NF-κB-mediated inflammation. Overall, our results highlight decursinol as a promising therapeutic candidate for inflammation-associated pregnancy complications.

## 1. Introduction

Pregnancy success relies on the precise orchestration of maternal physiological changes to support fetal development, with the placenta playing a central role in this process. The placenta facilitates nutrient and gas exchange, hormone production, and immune tolerance at the maternal–fetal interface. Proper placental development requires trophoblast cell invasion into the maternal decidua to ensure adequate uteroplacental blood flow [1]. Disruptions in placental functions are implicated in major pregnancy complications, including preeclampsia, fetal growth restriction (FGR), and preterm birth, which are the key causes of maternal and neonatal morbidity and mortality worldwide [2,3,4]. Maternal inflammation is the primary driver of placental dysfunction. Inflammatory stimuli, such as bacterial lipopolysaccharides (LPSs), create a hostile uterine environment by activating various signaling cascades, such as the Toll-like receptor 4 pathway [5,6]. This leads to the activation of transcription factors, such as nuclear factor (NF)-κB, which orchestrates the expression of several pro-inflammatory cytokines and provides a critical priming signal for the assembly of intracellular inflammasome complexes [7].

Upon activation, NOD-like receptor family pyrin domain-containing 3 (NLRP3) inflammasome, a multi-protein platform, drives the maturation and secretion of potent pro-inflammatory cytokines, such as interleukin (IL)-1β, via caspase-1 activation [8]. Aberrant activation of the NLRP3 inflammasome in placental tissues is increasingly linked to various pregnancy complications. In the placental environment, NLRP3 activation contributes to trophoblast dysfunction and impaired invasion, ultimately compromising the maternal–fetal interface integrity [9,10,11]. Mitochondrial dysfunction and subsequent mitochondrial reactive oxygen species (mtROS) production are the key upstream triggers for NLRP3 inflammasome assembly. This places mitochondrial homeostasis at a critical nexus, linking environmental stressors, such as infections, to placental pathology-driving inflammatory pathways [12,13].

Considering the central role of inflammation in various pregnancy complications, therapeutic strategies to modulate inflammatory pathways are of great interest. Decursinol, a coumarin derivative isolated from the roots of *Angelica gigas* Nakai, exerts various pharmacological effects, including antioxidant, anti-inflammatory, and neuroprotective effects [14,15,16]. However, its potential protective effects against inflammation-driven placental dysfunction and adverse pregnancy outcomes remain unclear.

In this study, we aimed to elucidate the protective action mechanisms of decursinol against LPS-induced placental inflammation and trophoblast dysfunction. We investigated the effects of decursinol using an in vitro model of human trophoblast cells and in vivo mouse model of LPS-induced pregnancy complications. Notably, decursinol protected against LPS-induced fetal and placental defects by suppressing the NF-κB and NLRP3 inflammasome signaling pathways. Mechanistically, decursinol mitigated LPS-induced impairment of mitochondrial homeostasis and reduced mtROS production, thereby attenuating inflammasome activation. Our findings highlight decursinol as a potential therapeutic candidate for inflammation-driven pregnancy complications.

## 2. Materials and Methods

### 2.1. Cell Culture and Reagent

Sw.71 human first-trimester trophoblast cell line (gifted by Dr. Gil Mor, Yale University School of Medicine, New Haven, CT, USA) was cultured in Dulbecco’s modified Eagle’s medium (Welgene, Gyeongsan, Republic of Korea) supplemented with 10% fetal bovine serum (Welgene) and 1% penicillin–streptomycin (100 U/mL penicillin and 100 µg/mL streptomycin) and maintained at 37 °C in a humidified atmosphere of 5% CO_2_. Decursinol was purchased from Cayman Chemical (Ann Arbor, MI, USA) and dissolved in dimethyl sulfoxide to prepare a 5-mM stock solution. LPS from Escherichia coli O127:B8 (Sigma-Aldrich, St. Louis, MO, USA) and ATP (Sigma-Aldrich) were used to stimulate NLRP3 inflammasome activation. Mito-TEMPO (Santa Cruz Biotechnology, Santa Cruz, CA, USA) was used as an mtROS scavenger in mechanistic assays. For experiments assessing NF-κB signaling, Sw.71 cells were stimulated with LPS alone for the indicated time periods. For experiments evaluating NLRP3 inflammasome activation, cells were first treated with LPS for 24 h and then exposed to ATP for the final 45 min to induce inflammasome activation.

### 2.2. Cell Viability Assay

To assess the cytotoxicity of decursinol, Sw.71 cells were seeded in a 96-well plate (1 × 10^4^ cells/well) and treated with increasing concentrations of decursinol (5–60 µM) for 24 and 48 h. Cell viability was analyzed using a WST-8 assay kit (Biomax, Seoul, Republic of Korea), according to the manufacturer’s instructions. Absorbance was measured at 450 nm using a microplate reader (Epoch BioTek, Winooski, VT, USA).

### 2.3. Immunoblotting Analysis

The cells and placental tissues were lysed using the radioimmunoprecipitation assay buffer containing protease and phosphatase inhibitors (Roche, Basel, Switzerland). Protein concentrations were determined via bicinchoninic acid protein assay (Thermo Fisher Scientific, Waltham, MA, USA). Proteins were separated via SDS-PAGE and transferred to PVDF membranes (Millipore, Burlington, MA, USA). The membranes were blocked with 5% non-fat milk in Tris-buffered saline with Tween-20 and probed overnight with primary antibodies against NLRP3 (13158; Cell Signaling Technology, Danvers, MA, USA), caspase-1 (2225; Cell Signaling Technology), ASC (sc-514414; Santa Cruz Biotechnology), p65 (8242; Cell Signaling Technology), phospho-p65 (3033; Cell Signaling Technology), IκBα (4814; Cell Signaling Technology), phospho-IκBα (2859; Cell Signaling Technology), N-cadherin (13116; Cell Signaling Technology), Snail (3879; Cell Signaling Technology), vimentin (5741; Cell Signaling Technology), β-actin (JLA20; Developmental Studies Hybridoma Bank, Iowa City, IA, USA), and GAPDH (2G7; Developmental Studies Hybridoma Bank) at 4 °C. After incubation with horseradish peroxidase-conjugated secondary antibodies, protein bands were visualized using enhanced chemiluminescence (ECL; Thermo Fisher Scientific) and quantified using the ImageJ software version 1.54t (National Institutes of Health, Bethesda, MD, USA).

### 2.4. Reverse Transcription-Quantitative Polymerase Chain Reaction (RT-qPCR)

Total RNA was isolated from the cells and placental tissues using the TRIzol reagent (Takara, Shiga, Japan) and reverse-transcribed using a reverse transcription kit (BioFact, Seoul, Republic of Korea). Subsequently, RT-qPCR was performed using the SYBR Green Master Mix (BioFact) on the QuantStudio 3 Real-Time PCR System (Life Technologies, Carlsbad, CA, USA). Relative gene expression was calculated using the 2−ΔΔCt method, with cyclophilin A as an internal control. All primer sequences are listed in Table S1.

### 2.5. Enzyme-Linked Immunosorbent Assay (ELISA)

Sw.71 cell culture supernatants were collected, centrifuged to remove any debris, and assayed for human IL-1β levels using the DuoSet ELISA Development Kit (R&D Systems, Minneapolis, MN, USA), according to the manufacturer’s protocol. Absorbance was measured at 450 nm using the microplate reader.

### 2.6. Trophoblast Invasion Assay

Cell invasion was assessed using Matrigel-coated transwell chambers (8-µm pore size; Corning Inc, Corning, NY, USA). Sw.71 cells were seeded in a serum-free medium in the upper chamber, whereas the lower chamber contained a medium with 10% fetal bovine serum. After 24 h, non-invading cells were removed, and invading cells on the lower surface of the membrane were fixed, stained with crystal violet (Sigma-Aldrich), and counted under a light microscope.

### 2.7. Mitochondrial Function and ROS Assays

Mitochondrial integrity was evaluated by staining the cells with MitoTracker Red (M7512; Thermo Fisher Scientific). After treatment, Sw.71 cells were incubated with MitoTracker Red according to the manufacturer’s instructions, washed with phosphate-buffered saline, and imaged using a fluorescence microscope. Mitochondrial morphology was quantified from microscopic images using ImageJ/Fiji software version 1.54p: images were thresholded and skeletonized, and the number of individual mitochondrial particles per cell and the mean mitochondrial branch length were measured. Values were normalized to those of the control group. Mitochondrial membrane potential (Δψm) was assessed using the JC-1 dye (T3168; Thermo Fisher Scientific). After staining, fluorescence was measured using a fluorescence microplate reader (Infinite M200; Tecan, Männedorf, Switzerland), and Δψm was expressed as the ratio of red fluorescence to green fluorescence. The values were normalized to the control group. Mitochondrial superoxide production and total intracellular ROS levels were detected using MitoSOX Red (M36008; Thermo Fisher Scientific) and DCFDA (D399; Thermo Fisher Scientific), respectively. The fluorescence signals for these assays were measured using a fluorescence microplate reader at their respective excitation/emission wavelengths (MitoSOX: 510/580 nm; DCFDA: 485/535 nm). For all fluorescence-based assays, background fluorescence was subtracted before normalization. Data are presented as relative fluorescence intensity compared with the control group.

### 2.8. Animal Experiments

All animal experiments were approved by the Institutional Animal Care and Use Committee of Konyang University (approval no. P-19-30-A-01) and were conducted in accordance with the approved guidelines. Pregnant C57BL/6 mice (8–10 weeks old) were randomly assigned to the control (*n* = 3 dams), LPS (*n* = 4 dams), and LPS + decursinol (*n* = 4 dams) groups. In the decursinol-treated group, mice received a sequential two-dose regimen consisting of a 1.5 mg/kg intraperitoneal injection on gestational day (GD) 16.5, followed by a 3 mg/kg injection on GD 17.5. LPS was administered intraperitoneally at 200 µg/kg on GD 17.5 to induce acute placental inflammation and fetal–placental injury [10]. The decursinol dosing schedule was selected to provide compound exposure before and during the LPS-induced inflammatory challenge while minimizing repeated handling and injection-related stress in pregnant mice [17,18]. Fetal and placental tissues were collected on GD 18.5 for histological, molecular, and biochemical analyses. A total of 17–22 fetuses were obtained from all litters and included in the analysis. The sex of fetuses was not determined in this study.

### 2.9. Histology and Immunohistochemistry

Placental tissues were fixed with 10% neutral buffered formalin, embedded in paraffin, sectioned (5 µm), and stained with hematoxylin and eosin or periodic acid–Schiff for histological evaluation. For immunohistochemistry, the sections were subjected to antigen retrieval, blocked with 5% bovine serum albumin, and incubated with antibodies against NLRP3 (BS-10021R; Thermo Fisher Scientific) and IL-1β (12242; Cell Signaling Technology). Subsequent visualization was achieved using horseradish peroxidase-conjugated secondary antibodies and diaminobenzidine substrate (Sigma-Aldrich). Images were acquired using a light microscope. For quantitative analysis of immunohistochemistry, staining intensity for NLRP3 and IL-1β was quantified using ImageJ software.

### 2.10. Statistical Analysis

For cell-based experiments, at least three independent biological replicates were analyzed per condition. Each data point represents one independent biological replicate. A formal a priori power analysis was not performed for the animal experiments. The sample size for animal experiments was determined empirically, based on the range of group sizes reported in previous studies using the same LPS-induced pregnancy complication model [10,19], while minimizing animal use. Data are represented as the mean ± standard error of the mean. Statistical analyses were performed using GraphPad Prism software version 8 (GraphPad Software, San Diego, CA, USA). Statistical significance was determined via one-way analysis of variance, followed by Fisher’s least significant difference post-hoc test, or two-tailed Student’s *t*-test, where appropriate. Statistical significance was set at *p* < 0.05.

## 3. Results

### 3.1. Decursinol Suppresses LPS-Induced NF-κB Signaling and NLRP3 Inflammasome Activation in Trophoblasts

**Figure 1:**
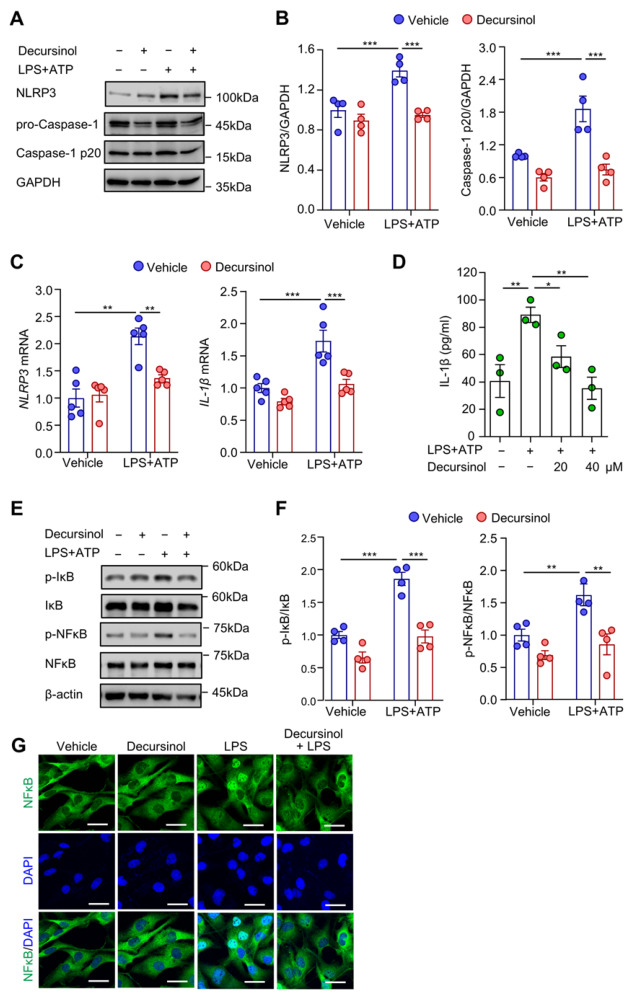
Decursinol inhibits lipopolysaccharide (LPS)-induced NOD-like receptor family pyrin domain-containing 3 (NLRP3) inflammasome activation and nuclear factor (NF)-κB/p65 signaling in trophoblasts. (**A**,**B**) Immunoblots of NLRP3 and caspase-1 in the lysates of Sw.71 cells treated with 20 µM decursinol for 30 min, followed by 1 µg/mL LPS for 24 h and 5 mM ATP for the final 45 min. (**C**) Relative mRNA levels of NLRP3, ASC, interleukin (IL)-1β, and caspase-1 in the lysates of Sw.71 cells. (**D**) IL-1β levels in Sw.71 cell culture supernatants. Sw.71 cells were treated with 20 µM decursinol for 30 min, followed by 1 µg/mL LPS for 24 h and 5 mM ATP for the final 45 min. IL-1β concentrations were quantified via enzyme-linked immunosorbent assay (ELISA). (**E**,**F**) Immunoblots of p-IκBα, IκBα, p-p65, and p65 in the lysates of Sw.71 cells treated with 1 µg/mL LPS or vehicle for 1 h in the presence or absence of 20 µM decursinol. β-actin or glyceraldehyde-3-phosphate dehydrogenase (GAPDH) served as a loading control. Band intensities were quantified and normalized to the control band intensities. (**G**) Immunofluorescence staining for p65 in Sw.71 cells after the indicated treatments. Nuclei were stained with Hoechst 33342. Scale bars, 20 µm. Data are represented as the mean ± SEM of independent biological replicates. * *p* < 0.05, ** *p* < 0.01, and *** *p* < 0.001.

To assess its safety profile, we evaluated the potential cytotoxicity of decursinol using Sw.71 human trophoblast cells. Sw.71 cells were exposed to various concentrations of decursinol (5–60 μM) for 24 and 48 h. WST-8 assay revealed no significant cytotoxicity at therapeutic concentrations up to 40 µM at both time points, indicating a safe dose range for subsequent in vitro experiments (Figure S1). To investigate the anti-inflammatory effects of decursinol, we examined its impact on the NLRP3 inflammasome pathway in Sw.71 cells. Immunoblotting analysis revealed that LPS robustly increased, whereas decursinol markedly decreased the NLRP3 and cleaved caspase-1 expression levels (Figure 1A,B). Consistent with these protein-level changes, RT-qPCR analysis indicated that decursinol markedly reduced the LPS-induced NLRP3 and IL-1β mRNA transcription levels (Figure 1C). Furthermore, ELISA of the cell culture supernatant indicated that decursinol significantly decreased mature IL-1β secretion (Figure 1D). As NF-κB signaling drives the transcriptional priming of the NLRP3 inflammasome, we assessed its activation status by evaluating the phosphorylation of the p65 subunit (RelA), which is the active component of the heterodimeric NF-κB complex. LPS stimulation led to the robust phosphorylation of IκBα at Ser32 and p65 at Ser536, both mediated by upstream IKKβ activity as part of the canonical NF-κB activation cascade. However, pretreatment with decursinol effectively inhibited the LPS-induced phosphorylation of both proteins (Figure 1E,F). Consistently, immunofluorescence staining revealed that decursinol prevented the nuclear translocation of p65 in response to LPS (Figure 1G). These results suggest that decursinol suppresses LPS-induced NLRP3 inflammasome activation alongside inhibition of NF-κB/p65 signaling, consistent with NF-κB-driven priming of the inflammasome pathway.

### 3.2. Decursinol Restores the Trophoblast Invasive Capacity Impaired by LPS

**Figure 2:**
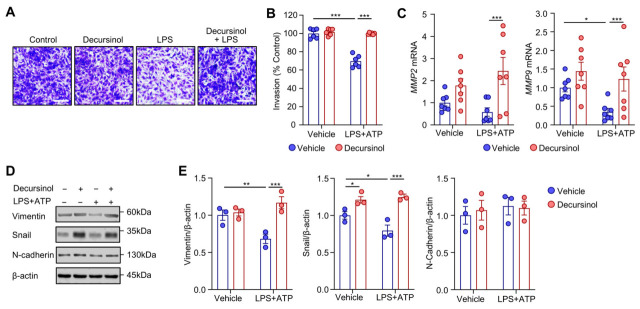
Decursinol restores the trophoblast invasive capacity impaired by LPS. (**A**,**B**) Representative images and quantification of Matrigel invasion by Sw.71 cells treated with 20 µM decursinol for 30 min, followed by 1 µg/mL LPS for 18 h and 5 mM ATP for the final 45 min. (**C**) Relative mRNA levels of matrix metalloproteinase (MMP)-2 and MMP-9 in Sw.71 cell lysates. (**D**,**E**) Immunoblots of N-cadherin, Snail, and vimentin in the lysates of Sw.71 cells treated with 20 µM decursinol for 30 min, followed by 1 µg/mL LPS for 24 h and 5 mM ATP for the final 45 min. β-actin served as a loading control. Band intensities were quantified and normalized to the control band intensities. Data are represented as the mean ± SEM of independent biological replicates. * *p* < 0.05, ** *p* < 0.01, and *** *p* < 0.001.

Inflammation impairs the invasive function of trophoblasts, which is critical for successful placentation [10]. Therefore, we investigated whether decursinol rescues trophoblast invasion inhibited by LPS. Matrigel invasion assays showed that LPS markedly reduced, whereas decursinol significantly restored the invasive capacity of Sw.71 cells (Figure 2A,B). Supporting these functional changes, RT-qPCR analysis revealed that decursinol reversed the LPS-induced downregulation of matrix metalloproteinase-2 and -9 levels (Figure 2C). Additionally, immunoblotting analysis indicated that decursinol preserved the mesenchymal marker vimentin and Snail expression levels, which were reduced by LPS (Figure 2D,E). Together, these data suggest that decursinol protects the trophoblast invasive capacity against LPS-induced dysfunction.

### 3.3. Decursinol Improves the Fetal and Placental Outcomes of LPS-Treated Mice

**Figure 3:**
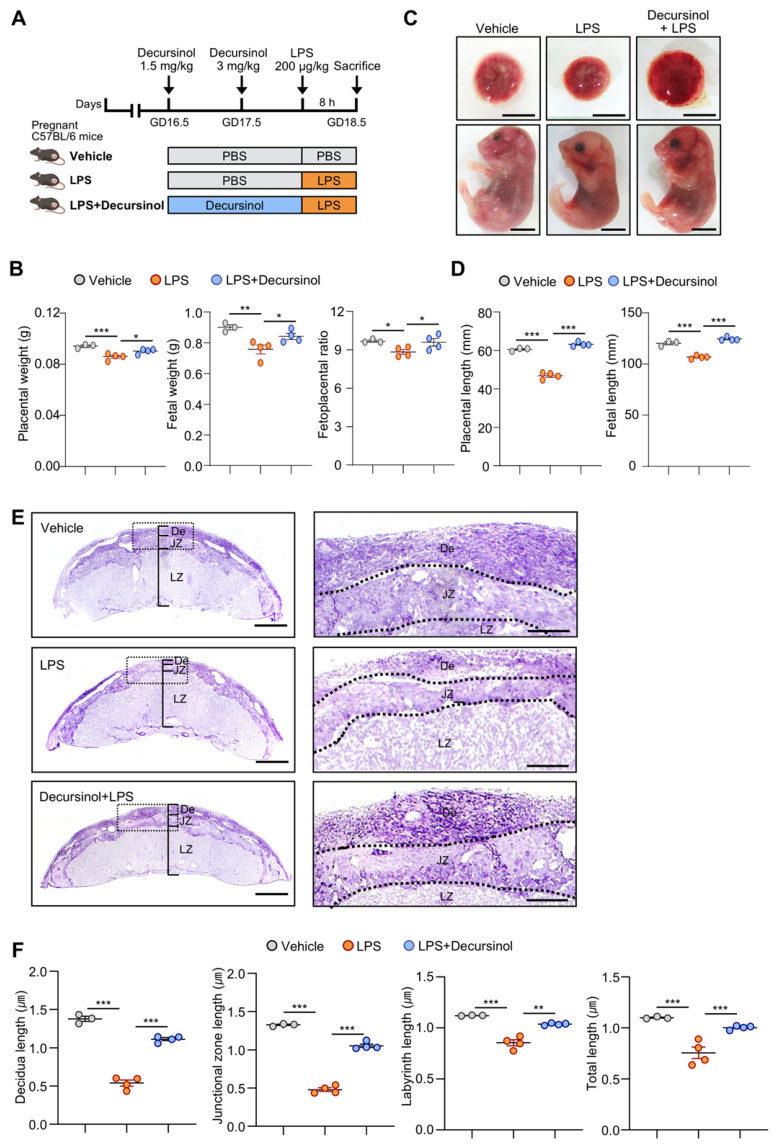
Effect of decursinol on gross placental morphology in LPS-treated pregnant mice. (**A**) Experimental timeline of the treatments administered to pregnant mice. (**B**) Representative images of the fetus and placenta of the control, LPS-treated, and LPS + decursinol-treated groups. Fetal and placental samples were collected on gestational day 18.5. Scale bars, 0.5 cm. (**C**,**D**) Average fetal weight, fetal length, placental weight, placental length, and fetoplacental weight ratio in control, LPS-treated, and LPS + decursinol-treated groups. (**E**,**F**) Periodic acid–Schiff (PAS) staining of the placental tissues of the control, LPS-treated, and LPS + decursinol-treated groups. Decidua (De), labyrinth (LZ), and junctional zone (JZ) lengths in the midline section were quantified. Areas highlighted by boxes are shown at higher magnification in the adjacent panels. The dashed lines delineate the boundaries between the decidua, junctional zone, and laby-rinth zone. Scale bars, 1 mm (main panels) and 250 µm (insets). Data are represented as the mean ± SEM (*n* = 3–4 dams per group). * *p* < 0.05, ** *p* < 0.01, and *** *p* < 0.001.

To evaluate the protective effects of decursinol in vivo, we used a mouse model of LPS-induced pregnancy complications (Figure 3A). On GD 18.5, LPS exposure significantly reduced the fetal and placental weights and lengths compared to those in the control group. However, decursinol administration significantly improved the fetal and placental growth parameters (Figure 3B–D). The fetoplacental ratio was significantly reduced in the LPS-treated group compared to controls, indicating relative placental insufficiency; decursinol treatment partially restored this ratio toward control levels (Figure 3C). Histological examination via periodic acid–Schiff staining revealed that LPS treatment markedly disrupted normal placental architecture. In the LPS-treated group, the placental layers appeared disorganized, with a noticeable reduction in the thickness and continuity of the decidua, labyrinth zone, and junctional zone compared with those in the control group. In particular, the labyrinth zone, which is essential for maternal–fetal exchange, exhibited structural narrowing and loss of normal tissue organization, whereas the junctional zone showed reduced expansion and impaired structural integrity. In contrast, decursinol treatment partially restored the overall placental architecture, preserving the distinct boundaries and relative thickness of the decidua, labyrinth zone, and junctional zone. Quantitative analysis revealed significantly reduced decidual, labyrinth, and junctional zone lengths in LPS-treated groups, which were partially restored by decursinol (Figure 3E,F). These findings suggest that decursinol protects the placental structural integrity under inflammatory conditions.

### 3.4. Decursinol Attenuates Placental Inflammation and NLRP3 Inflammasome Activation In Vivo

**Figure 4:**
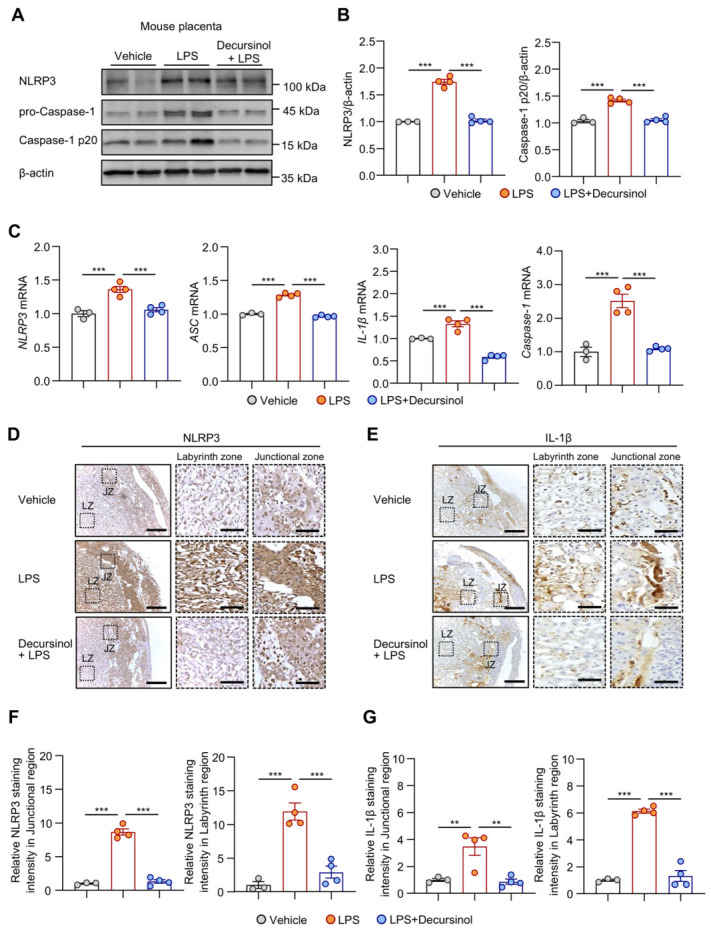
Decursinol suppresses placental NLRP3 inflammasome activation in vivo. (**A**,**B**) Immunoblots of NLRP3 and caspase-1 in the placental tissue lysates of the control, LPS-treated, and LPS + decursinol-treated groups. GAPDH served as a loading control. Band intensities were quantified and normalized to the control band intensities. (**C**) Relative mRNA levels of NLRP3, IL-1β, caspase-1, and apoptosis-associated speck-like protein containing a caspase-recruitment domain (ASC) in the placental tissue lysates of the control, LPS-treated, and LPS + decursinol-treated groups. (**D**,**E**) Immunohistochemical staining for NLRP3 and IL-1β in the placental tissues of the control, LPS-treated, and LPS + decursinol-treated groups. Nuclei were visualized using hematoxylin. Areas highlighted by boxes are shown at higher magnification in the adjacent panels. LZ and JZ indicate the labyrinth and junctional zones, respectively. Scale bars, 200 µm and 100 µm (insets). (**F**,**G**) Quantification of the NLRP3 and IL-1β immunohistochemical staining intensity in placental tissues. Data are represented as the mean ± SEM (*n* = 3–4 dams per group). ** *p* < 0.01, and *** *p* < 0.001.

**Figure 5:**
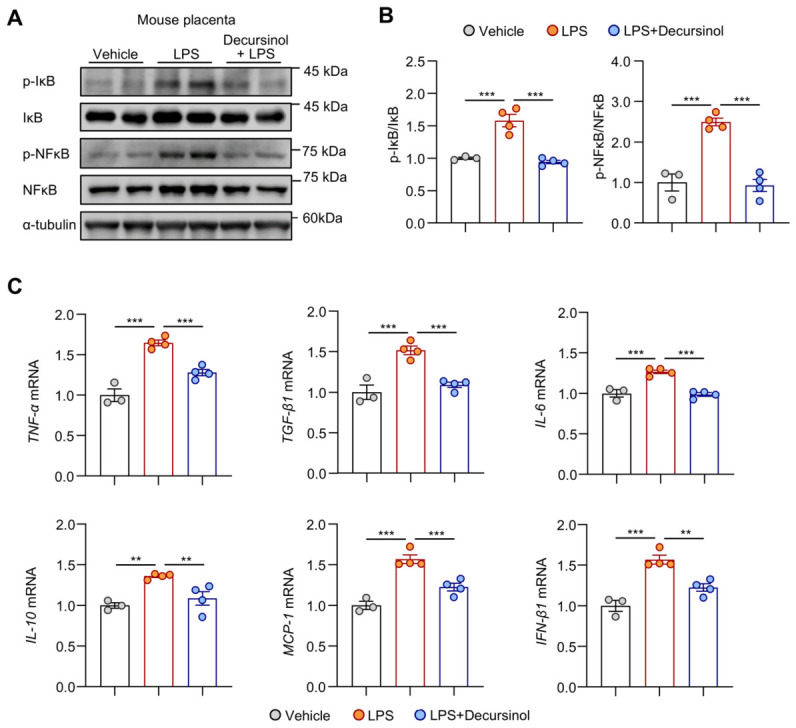
Decursinol attenuates placental inflammation in vivo. (**A**,**B**) Immunoblots of p-IκBα, IκBα, p-p65, and p65 in the placental tissue lysates of the control, LPS-treated, and LPS + decursinol-treated groups. β-actin served as a loading control. Band intensities were quantified and normalized to the control band intensities. (**C**) Relative mRNA levels of tumor necrosis factor (TNF)-α, transforming growth factor (TGF)-β1, IL-6, IL-10, interferon (IFN)-β, and monocyte chemotactic protein (MCP)-1 in the placental tissue lysates of the control, LPS-treated, and LPS + decursinol-treated groups. Data are represented as the mean ± SEM (*n* = 3–4 dams per group). ** *p* < 0.01, and *** *p* < 0.001.

We further assessed whether the protective effects of decursinol in vivo are associated with the suppression of the NLRP3 inflammasome and NF-κB pathways in the placenta. In the placental tissues of LPS-treated mice, we observed markedly elevated NLRP3 and cleaved caspase-1 protein levels, which were significantly decreased by decursinol (Figure 4A,B). RT-qPCR analysis confirmed these findings, showing that decursinol attenuated the LPS-induced upregulation of NLRP3, ASC, IL-1β, and caspase-1 mRNA expression levels (Figure 4C). Immunohistochemical staining revealed strong NLRP3 and IL-1β staining in the labyrinth and junctional zones of LPS-treated placentas, which was significantly reduced by decursinol (Figure 4D,E). Quantitative analysis of these images confirmed that the positive staining areas for both NLRP3 and IL-1β were significantly higher in the LPS-treated group compared to the control group, whereas decursinol administration significantly attenuated this increase (Figure 4F,G). Simultaneously, we examined NF-κB signaling and downstream inflammatory cytokine expression levels in the placental tissues. Immunoblotting analysis confirmed that LPS activated placental NF-κB signaling, evidenced by the increased phosphorylation levels of IκBα and p65. However, decursinol significantly reduced these activation marker levels (Figure 5A,B). Placental cytokine profiling via RT-qPCR revealed that LPS upregulated the levels of multiple inflammatory cytokines, including tumor necrosis factor-α, transforming growth factor-β1, IL-6, IL-10, interferon-β, and monocyte chemotactic protein-1. However, decursinol significantly reduced the mRNA levels of these inflammatory cytokines (Figure 5C). Collectively, these findings confirmed that decursinol exerted protective effects in vivo by attenuating placental inflammation via inhibition of the NLRP3 inflammasome and NF-κB signaling pathways.

### 3.5. Decursinol Suppresses Inflammasome Activation by Attenuating LPS-Induced Mitochondrial Stress

**Figure 6:**
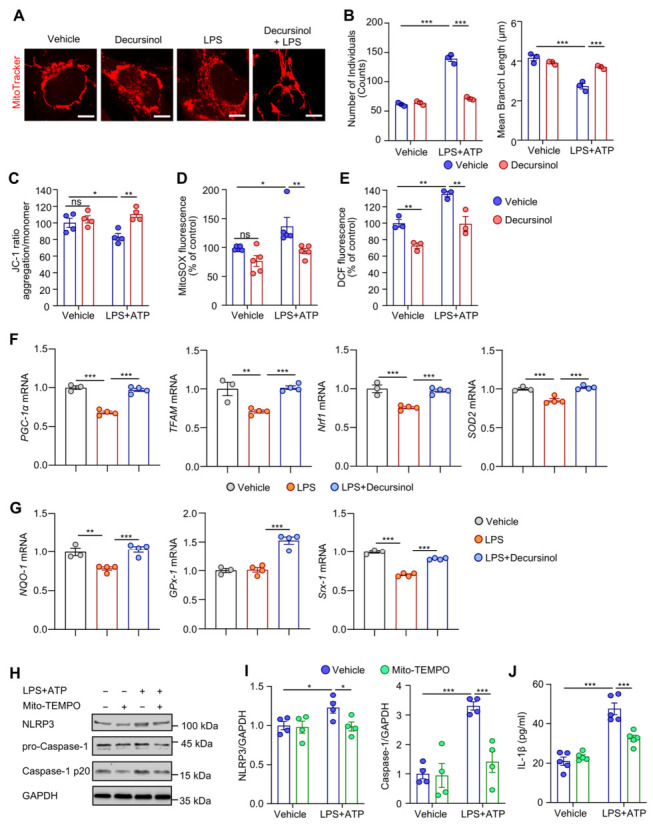
Decursinol mitigates LPS-induced mitochondrial homeostasis and suppresses NLRP3 inflammasome activation. (**A**) MitoTracker Red staining of Sw.71 cells treated with 20 µM decursinol for 30 min, followed by 1 µg/mL LPS for 6 h. Scale bars, 10 μm. (**B**) Number of individual mitochondrial particles per cell and mean mitochondrial branch length, quantified from skeletonized MitoTracker Red images. (**C**–**E**) Quantitative analysis of mitochondrial membrane potential (JC-1), mitochondrial superoxide (MitoSOX Red), and intracellular ROS (DCFDA) measured using a fluorescence microplate reader. (**F**,**G**) Relative mRNA levels of mitochondrial biogenesis-related genes (PGC-1α, TFAM, Nrf1, and SOD-2) and antioxidant genes (NQO-1, Srx-1, Gpx-1) in the placental tissue lysates of the control, LPS-treated, and LPS + decursinol-treated groups (*n* = 3–4 dams per group). (**H**,**I**) Immunoblots of NLRP3 and caspase-1 in the lysates of Sw.71 cells treated with 100 µM Mito-TEMPO for 30 min, followed by 1 µg/mL LPS for 24 h and 5 mM ATP for the final 45 min. GAPDH served as a loading control. Band intensities were quantified and normalized to the control band intensities. (**J**) Human IL-1β levels in the cell culture supernatants of Sw.71 cells treated with 100 µM Mito-TEMPO for 30 min, followed by 1 µg/mL LPS for 24 h and 5 mM ATP for the final 45 min, measured via ELISA. For panels (**B**–**E**,**I**,**J**), data are represented as the mean ± SEM of independent biological replicates. For panels (**F**,**G**), data are represented as the mean ± SEM (*n* = 3–4 dams per group). * *p* < 0.05, ** *p* < 0.01, and *** *p* < 0.001. ns, not significant.

To elucidate the upstream mechanisms by which decursinol regulates the NLRP3 inflammasome, we investigated its role in mitochondrial homeostasis, a known trigger for inflammasome activation. Morphometric analysis of MitoTracker Red-stained cells revealed that LPS treatment significantly increased the number of individual mitochondrial particles per cell and significantly decreased the mean mitochondrial branch length compared to the control group, consistent with a shift toward a fragmented mitochondrial phenotype. Decursinol treatment significantly decreased the number of individual particles and significantly increased the mean branch length relative to the LPS-treated group, indicating partial restoration of a more interconnected mitochondrial network (Figure 6A,B). Moreover, JC-1 assay indicated that LPS significantly decreased the mitochondrial membrane potential, which was preserved by decursinol (Figure 6C). Assessment of mitochondrial oxidative stress using MitoSOX revealed increased mitochondrial superoxide production following LPS treatment; however, this effect was attenuated by decursinol (Figure 6D). Similarly, DCFDA assay revealed increased total cellular ROS levels following LPS treatment; however, this effect was attenuated by decursinol (Figure 6E). RT-qPCR analysis revealed that decursinol upregulated the placental expression levels of mitochondrial biogenesis-related genes (peroxisome proliferator-activated receptor-γ coactivator-1α, mitochondrial transcription factor A, nuclear respiratory factor 1, and superoxide dismutase 2) in vivo (Figure 6F). Additionally, decursinol restored the expression levels of several antioxidant genes (NAD(P)H:quinone oxidoreductase-1, sulfiredoxin-1, and glutathione peroxidase-1) in the placenta (Figure 6G). To assess the mechanistic link between mtROS and inflammasome activation, we used Mito-TEMPO, a mitochondria-targeted antioxidant. Similarly to decursinol, Mito-TEMPO reduced the LPS-induced NLRP3 and cleaved caspase-1 expression levels (Figure 6H,I) and decreased IL-1β secretion (Figure 6J). Furthermore, Mito-TEMPO attenuated NF-κB activation, as evidenced by the reduced IκBα and p65 phosphorylation levels (Figure S2). These results support an association between mitochondrial ROS regulation and inflammatory signaling, and indicate that LPS induces mitochondrial injury-related changes that are attenuated by decursinol.

## 4. Discussion

Maternal inflammation-driven adverse pregnancy outcomes, such as FGR, pose a significant clinical challenge with limited therapeutic options. This study provides compelling evidence that decursinol effectively counteracts the detrimental effects of inflammation on pregnancy. Notably, decursinol ameliorated LPS-induced FGR and placental defects in a mouse model. Mechanistically, this protection was mediated by the suppression of the NF-κB signaling pathway and subsequent inhibition of NLRP3 inflammasome activation in vitro and in vivo. Furthermore, our findings identified an upstream mechanism for these anti-inflammatory effects involving the preservation of mitochondrial homeostasis and attenuation of oxidative stress in trophoblasts.

The placenta is highly vulnerable to inflammatory insults disrupting maternal–fetal immune tolerance and compromising pregnancy [20,21]. Consistent with previous reports implicating excessive activation of the NLRP3 inflammasome and NF-κB pathways in pregnancy complications [22,23], this study found that LPS exposure robustly induced these pathways in trophoblasts and placenta, leading to elevated pro-inflammatory cytokine levels and structural disruption. However, decursinol significantly attenuated these effects, suggesting that targeting both the inflammasome priming and activation signals is a promising strategy to prevent inflammation-driven pregnancy disorders.

Interestingly, our results also showed a significant increase in the mRNA levels of IL-10, IFN-β, and TGF-β1 in placental tissues following LPS administration. While these factors are generally associated with anti-inflammatory or regulatory functions, their upregulation during acute LPS-induced inflammation is consistent with the induction of a compensatory anti-inflammatory response [24,25]. IL-10 and TGF-β1 are often upregulated as part of a physiological negative feedback loop aimed at tempering the primary pro-inflammatory cascade and preventing excessive tissue-destructive responses [26]. Similarly, the increase in IFN-β expression reflects the activation of the TRIF-dependent arm of TLR4 signaling, which occurs in parallel with the MyD88-dependent pro-inflammatory pathway [27]. The fact that significant placental dysfunction and fetal loss still occurred in the LPS-treated group suggests that this endogenous regulatory response was overwhelmed by the magnitude of the inflammatory insult [28]. Decursinol treatment further modulated these expression profiles, potentially aiding in the restoration of placental homeostasis.

NF-κB signaling and mitochondrial homeostasis do not operate independently. After LPS stimulation, canonical NF-κB activation increases the expression of inflammatory mediators such as inducible nitric oxide synthase (iNOS) and cyclooxygenase-2 (COX-2). These mediators can increase reactive nitrogen and oxygen species, which may damage mitochondrial electron transport, reduce mitochondrial membrane potential, and promote mitochondrial fragmentation and mtROS production [29,30]. mtROS can then further enhance inflammatory signaling and promote NLRP3 inflammasome activation [31,32]. This creates a self-reinforcing cycle of inflammation and mitochondrial stress. In our study, decursinol may reduce this cycle by suppressing inflammatory signaling and preserving mitochondrial function. However, the link between mtROS and NF-κB/NLRP3 activation in our model rests on pharmacological evidence using Mito-TEMPO. Future studies using genetic approaches, such as SOD2 overexpression or siRNA-mediated knockdown of NLRP3 or p65, will be needed to define the causal order of these events more clearly.

Therapeutic potential of decursinol was also assessed in vivo. Decursinol administration not only reduced placental inflammation but also improved the fetal and placental weights and preserved the placental architecture in LPS-treated pregnant mice. The successful translation of our in vitro mechanistic findings to a preclinical animal model further supports decursinol as a promising candidate for clinical use. As a compound derived from a natural source with known anti-inflammatory properties [33], decursinol possibly exhibits a favorable safety profile for use during pregnancy, warranting further investigation.

Although the LPS-induced model is widely used to study inflammation-associated pregnancy complications [34,35], it has inherent limitations that must be carefully considered when interpreting the translational potential of our findings. LPS mainly produces an acute TLR4-driven innate immune response. For this reason, it is useful for studying NF-κB signaling, inflammasome activation, and the effects of anti-inflammatory compounds. However, human preeclampsia and FGR usually develop over time and involve several overlapping processes. These include poor trophoblast invasion, incomplete spiral artery remodeling in early pregnancy, placental ischemia and hypoxia, endothelial dysfunction, metabolic stress, and abnormal immune adaptation [3,36]. Our study cannot determine whether decursinol would remain effective in chronic, low-grade oxidative stress or inflammation. This is different from the rapid and strong inflammatory response caused by LPS. Recent reports have also pointed out that acute inflammation models do not fully reproduce the long-term placental dysfunction seen in preeclampsia and FGR [37,38]. These disorders involve persistent oxidative stress, repeated ischemia–reperfusion injury, and prolonged immune dysregulation, which are not reproduced by a single LPS injection. Therefore, decursinol should be tested in additional chronic models. These may include the reduced uterine perfusion pressure model for preeclampsia or hypoxia-induced models of FGR. Such studies will be needed to determine whether the protective effects observed here also apply to the more complex pathology of human pregnancy disorders.

Despite these limitations, it is noteworthy that NF-κB/NLRP3 activation and mitochondrial oxidative stress have been documented in placental tissues from patients with preeclampsia and FGR [13,39,40,41], providing a biological rationale for further investigation in chronic models. In this context, the ability of decursinol to suppress inflammatory priming, reduce mitochondrial ROS, and preserve trophoblast invasive capacity suggests that it may target key pathogenic processes relevant to human placental disease. In particular, preservation of mitochondrial homeostasis may be clinically meaningful because mitochondrial dysfunction is increasingly recognized as a convergent mechanism linking inflammation, oxidative stress, and impaired placental function. Although the present findings are promising, further studies using primary human trophoblasts, patient-derived placental tissues, and pharmacokinetic and safety analyses will be required before decursinol can be considered a viable therapeutic candidate for use during pregnancy.

In the context of existing therapeutic approaches, decursinol presents several mechanistic advantages worth highlighting. Current options for inflammation-driven pregnancy complications are narrow. Corticosteroids broadly suppress inflammation but carry fetal risks with repeated use; nonsteroidal anti-inflammatory drugs are largely contraindicated after mid-pregnancy; low-dose aspirin targets platelet pathways rather than NLRP3 or mitochondrial oxidative stress; and broad-spectrum antioxidant trials with vitamins C and E have failed, likely because they do not reach the mitochondrial source of pathological ROS. Decursinol simultaneously targets these interconnected pathways by inhibiting initial NF-κB signaling, dampening NLRP3 inflammasome assembly, and sustaining mitochondrial health, while exhibiting an encouragingly low cytotoxicity profile at standard therapeutic concentrations [42,43]. However, pharmacokinetics during pregnancy, placental transfer, and teratogenicity have not been assessed, and clinical data are absent. These gaps must be addressed before any translational claims can be made. A further limitation is the developmental difference between the in vitro and in vivo models used in this study. Sw.71 cells are derived from first-trimester human trophoblasts and are useful for studying trophoblast inflammatory responses and invasive capacity [44]. In contrast, the mouse placental tissues were collected at late gestation after LPS exposure. Because trophoblast differentiation state, gene expression, and placental function change across gestation, these two models should be considered complementary rather than developmentally equivalent. Future studies using gestational stage-matched trophoblast models, primary human trophoblasts, placental explants, or stage-specific animal models will be needed to confirm whether the protective effects of decursinol are conserved across placental developmental stages.

The present study evaluated decursinol within a preventive paradigm, administered prior to and concurrently with LPS challenge, rather than after the onset of placental inflammation. This raises the question of how such a prophylactic strategy could realistically be translated into clinical use. Because the continuous administration of pharmacological therapies across a general pregnant population inherently introduces potential safety and developmental exposure risks, the clinical application of decursinol would be most appropriate as a highly selective prophylactic strategy. Specifically, it could be utilized to protect patients definitively identified as possessing an elevated risk for severe inflammatory placental disorders. This proposed clinical framework closely mirrors the current obstetric paradigm wherein low-dose aspirin is selectively prescribed as a preventative measure against preeclampsia in vulnerable maternal populations [45]. The current investigation did not evaluate the therapeutic potential of decursinol if administered subsequent to the onset of established inflammatory alterations. Consequently, determining whether the compound maintains its protective efficacy in a post-symptomatic or established disease context remains a critical objective for subsequent research endeavors.

## 5. Conclusions

**Figure 7:**
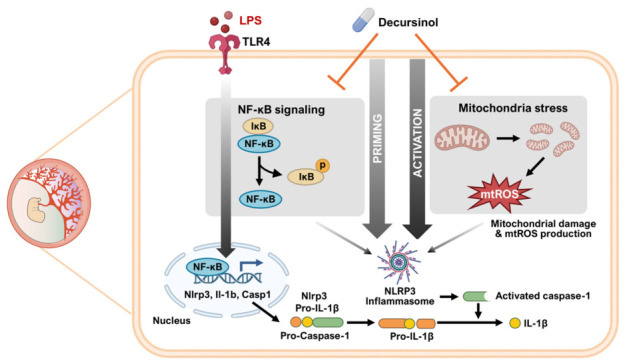
Schematic overview of the molecular mechanisms by which decursinol protects against LPS-induced placental inflammation. Arrows indicate activation or progression of signaling events, whereas inhibitory lines indicate suppressive effects.

In conclusion, this study demonstrates that decursinol attenuates LPS-induced placental inflammation and trophoblast dysfunction. These protective effects are likely mediated through the preservation of mitochondrial integrity and the subsequent inhibition of the NF-κB/NLRP3 inflammatory axis (Figure 7). These findings support decursinol as a candidate compound with anti-inflammatory and mitochondria-preserving effects in experimental models of placental inflammatory injury. While the acute nature of the LPS model limits the direct translation of these findings to chronic human syndromes, our results provide a comprehensive mechanistic basis for further investigating decursinol as a potential prophylactic intervention in pregnancies at a high risk of experiencing excessive placental inflammation and mitochondrial stress. Furthermore, advancing this clinical potential will depend upon future research to validate its therapeutic efficacy when administered following the onset of established pathological changes.

## Data Availability

The original contributions presented in this study are included in the article/Supplementary Materials. Further inquiries can be directed to the corresponding author.

## Supplementary Materials

The following supporting information can be downloaded at: https://www.mdpi.com/article/10.3390/cells15141309/s1, Table S1: List of primer pairs used for RT-qPCR analysis; Figure S1: In vitro cytotoxicity of decursinol in human trophoblast cells; Figure S2: Mito-TEMPO attenuates LPS-induced NF-kB/p65 activation in trophoblasts.
