# Supplementary material for: Decursinol Protects Against Lipopolysaccharide-Induced Placental Inflammation and Trophoblast Dysfunction via Mitochondrial Preservation and NLRP3 Inflammasome Inhibition
Source: Cells. 2026 Jul 22;15(14):1309. doi: 10.3390/cells15141309 (PMC13406846; doi:10.3390/cells15141309)
Supplement: Supplementary file 1 [file cells-15-01309-s001.zip › cells-4404918-supplementary.pdf]

Supplementary Material

Table S1. List of primer pairs used for RT-qPCR analysis.

Gene	Forward Primer (5`-3`)	Reverse Primer (5`-3`)
m_Cyclophilin A	GAGCTGTTTGCAGACAAAGTTC	CCCTGGCACATGAATCCTGG
m_IFN-β	GCACTGGGTGGAATGAGACT	AGTGGAGAGCAGTTGAGGAC
m_IL-6	TAGTCCTTCCTACCCCAATTTCC	TTGGTCCTTAGCCACTCCTTC
m_TNF-α	TCCCAGGTTCTCTTCAAGGGA	GGTGAGGAGCACGTAGTCGG
m_MCP-1	CATCCACGTGTTGGCTCA	GATCATCTTGCTGGTGAATGAGT
m_IL-10	GCTCTTACTGACTGGCATGAG	CGCAGCTCTAGGAGCATGTG
m_TGF-β1	CTCCCGTGGCTTCTAGTGC	GCCTTAGTTTGGACAGGATCTG
m_ASC	GAAGCTGACAGTGCAAC	GCCACAGCTCCAGACTCTTC
m_IL-1β	TCTTTGAAGTTGACGGACCC	TCTTTGAAGTTGACGGACCC
m_Caspase-1	AGATGGCACATTTCC AGGAC	GATCCTCCAGCAACTTC
m_NLRP3	AGCCTTCCAGGATCCTCTTC	CCTGGG CAGTTTCTT TC
m_PGC-1α	TATGGAGTGACATAGAGTGTGCT	CCACTTCAATCCACCCAGAAAG
m_TFAM	ATTCCGAAGTGTTTTTCCAGCA	TCTGAAAGTTTTGCATCTGGGT
m_Nrf1	AGCACGGAGTGACCCAAAC	TGTACGTGGCTACATGGACCT
m_SOD2	CAGACCTGCCTTACGACTATGG	CAGACCTGCCTTACGACTATGG
m_NQO-1	TTCTCTGGCCGATTCAGAG	GGCTGCTTGGAGCAAAATAG
m_Srx-1	GGAAGGAAGAAAGGAGATGGA	AGAGTTCAGGCTATGGGGATG
m_GPx-1	ACAGTCCACCGTGTATGCCTTC	CTCTTCATTCTTGCCATTCTCCTG
h_Cyclophilin A	GCAAAGTGAAAGAAGGCATGAA	CCATTCCTGGACCCAAAGC
h_NLRP3	GATCTTCGCTGCGATCAACAG	CGTGCATTATCTGAACCCCAC
h_IL-1β	AAAGAGGCACTGGCAGAA	AGCTCTGGCTTGTTCCTCAC
h_MMP2	GTCTGTGTTGTCCAGAGGCA	CTAGGCCAGCTGGTTGGTTC
h_MMP9	GTACTCGACCTGTACCAGCG	AGAAGCCCCACTTCTTGTCG

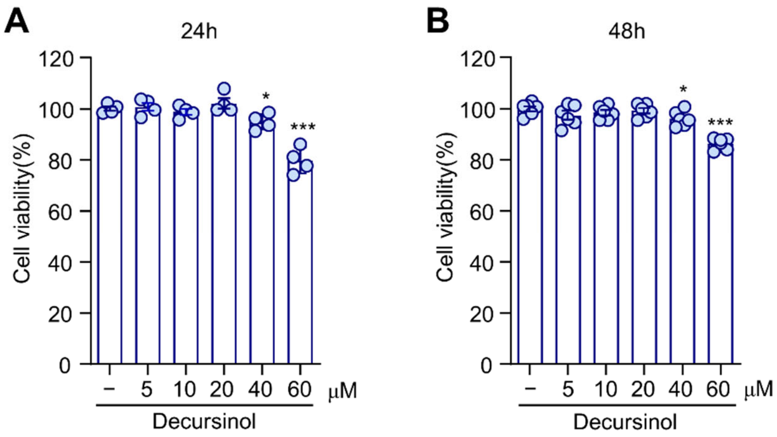

Figure S1. In vitro cytotoxicity of decursinol in human trophoblast cells. (A) S2.71 cells were treated with the indicated concentrations of decursinol for 24 (A) and 48 h (B). Cell viability was measured via WST-8 assay. Data are shown as means ± SEM. *$p < 0.05$; ***$p < 0.001$.

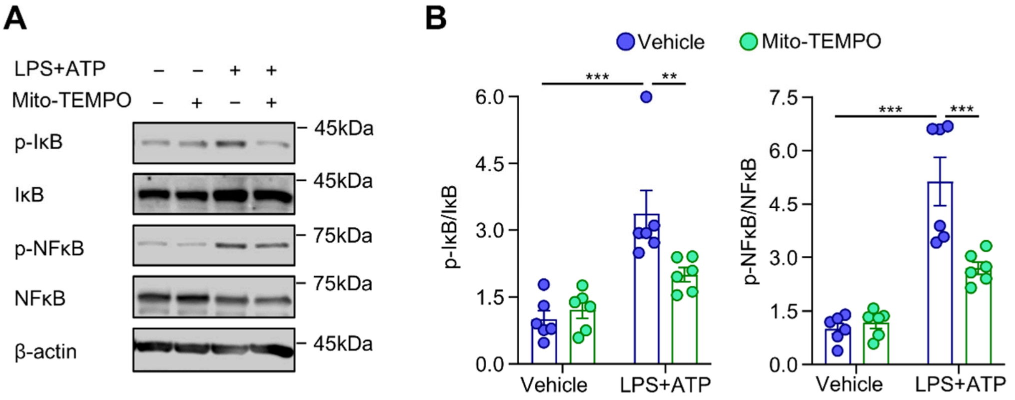

Figure S2. Mito-TEMPO attenuates LPS-induced NF-kB/p65 activation in trophoblasts. (A and B) Immunoblots of p-IκBα, IκBα, p-p65, and p65 in the lysates of Sw.71 cells treated with 100 μM Mito-TEMPO for 30 min followed by 1 μg/mL LPS for 24 h with 5 mM ATP for the last 45 min. β-actin served as a loading control. Band intensities were quantified and normalized to control band intensities. Data are shown as means ± SEM. **p < 0.01; ***p < 0.001.
